# Growth Patterns of Placental and Paraovarian Adrenocortical Heterotopias Are Different

**DOI:** 10.1155/2013/205692

**Published:** 2013-12-07

**Authors:** Hua Zhong, Bo Xu, Dorota A. Popiolek

**Affiliations:** ^1^Department of Pathology and Laboratory Medicine, Rutgers Robert Wood Johnson Medical School and Rutgers Cancer Institute of New Jersey, New Brunswick, NJ 08903, USA; ^2^Department of Pathology and Laboratory Medicine, Roswell Park Cancer Institute, Buffalo, NY 14263, USA; ^3^Department of Pathology and Laboratory Medicine, New York University School of Medicine, New York, NY 10016, USA

## Abstract

Two cases of adrenocortical heterotopia are reported. One is in a full-term placenta. The other is adjacent to the ovarian hilum of an adult. Both are incidental findings. Despite sharing similar histological and immunological features, they show different growth patterns. The literature is reviewed and adrenocortical heterotopias of different locations are compared. New hypotheses of its histogenesis are discussed.

## 1. Introduction

Adrenocortical heterotopia is usually an incidental finding in surgical pathology practice. It is often a solitary round micronodule comprising pure adrenocortical tissue with clinically insignificant function. The majority of heterotopic locations are closely related to the bilateral routes of gonadal descent. Distant heterotopia is, however, not uncommon and can be found in many major organ systems including the kidneys [1–3], lungs [4, 5], brain or spinal cord [6, 7], and liver [8–11]. Demographically, adrenocortical heterotopia can be found in men and women, pediatric and adult patients, and fetus or even placenta [12–16]. According to a general review by Dr. Jaffe of New York [17], adrenocortical heterotopia may be present in as many as 50% of postmortems on males of “new born or very young infant” and in 50% of normal rats at sexual maturity in the epididymal region if cautiously searched for. The majority of the heterotopias may become atrophic with maturation of the normal adrenal glands [17]. Herein, two cases of adrenocortical heterotopia are reported. One was within a full-term placenta. The other was embedded in the adipose tissue adjacent to the right ovarian hilum of an adult female patient. Their cytological and immunological features are similar while their architectural patterns are different.

## 2. Case A

**Figure 1:**
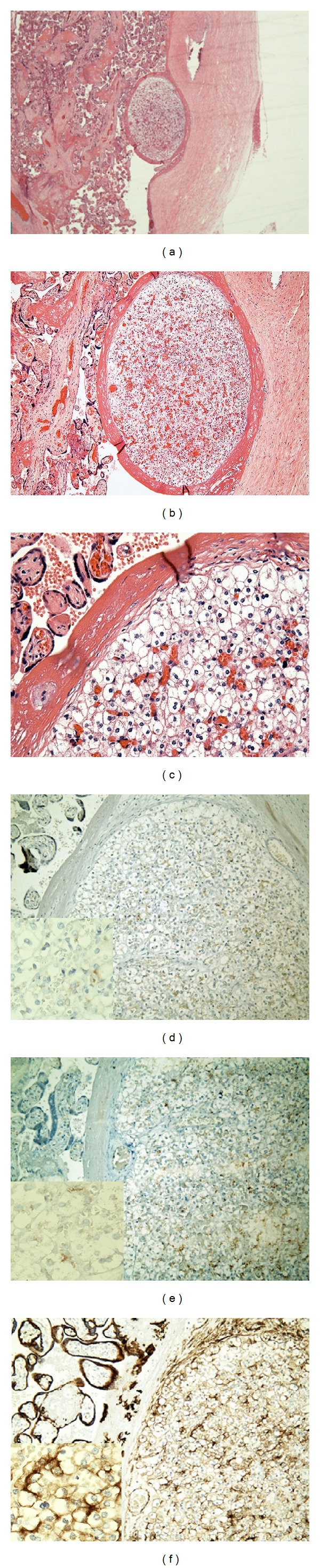
An adrenocortical heterotopia located in subchorionic space described in Case A. (a) Hematoxylin and eosin, ×20. (b) Hematoxylin and eosin, ×40. (c) Hematoxylin and eosin, ×200. (d) Immunohistochemical staining by using a monoclonal antibody against human inhibin alpha, ×100 (insert ×400). (e) Immunohistochemical staining by using a monoclonal antibody against human Melan-A, ×100 (insert ×400). (f) Immunohistochemical staining by using a monoclonal antibody against human Cam5.2, ×100 (insert ×400).

A 30-year-old woman, gravida 3, para 2, had a spontaneous vaginal delivery of a 40-week gestational age male infant. The mother's medical history was significant for abnormal glucose tolerance and a previous spontaneous abortion. The infant was 3,000 grams and 42 cm long with Apgar scores of 9. The development of the infant was normal at the one-year followup. The placenta received without fixatives was 560 grams and 19 × 19 × 2.5 cm. The umbilical cord with an eccentric placental insertion was grossly normal, and the fetal membranes were unremarkable. The cotyledons were red and complete. The placenta was sectioned at 0.5 cm intervals, and no gross abnormalities were noted on the cut surfaces. Two random full-thickness sections were taken for hematoxylin and eosin stain. Histologically, a 0.2 cm well-circumscribed subchorionic nodule was present and within a thick layer of fibrin-like materials that stimulated a pseudocapsule. A few small villi were embedded within this pseudocapsule. A definite fibrous capsule was not present. The nodule was compacted with oval or round cells intermingling with abundant fine vasculature. The cells had well-defined borders and a low nuclear/cytoplasm ratio. The cytoplasm showed clear spongy appearance that was partly clear and partly contained fine eosinophilic granules. Cells in the periphery appeared to have slightly less amount of eosinophilic granules and were less vascularized. Nuclei were small and frequently binucleated. Immunohistochemical stains were performed with appropriate controls. The cells within the subchorionic microscopic nodule were diffusely positive for inhibin alpha, Melan-A, and cytokeratin Cam5.2. Stains for inhibin alpha and Melan-A were cytoplasmic and granular and weak to moderate in intensity. The Cam5.2 stain was membranous and cytoplasmic and moderate to strong in intensity. All positive cells were diffusely distributed, and no specific patterns were seen. Both histological and immunological findings supported that the solitary nodule was heterotopic immature adrenocortical tissue (Figure 1). The overall growth architecture of the lesion had no pattern and did not appear to resemble either fetal or maturing adrenocortical tissue.

## 3. Case B

**Figure 2:**
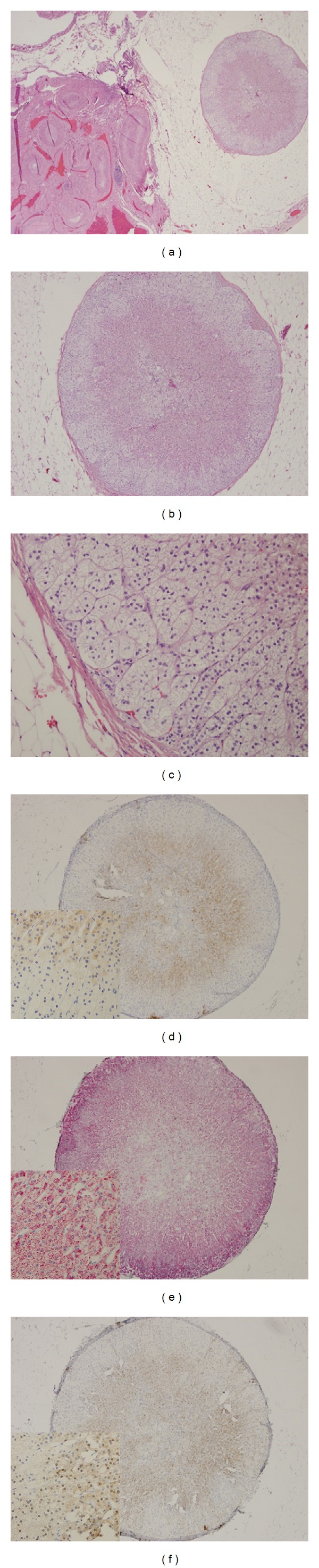
An adrenocortical heterotopia located adjacent to the ovarian hilum described in Case B. (a) Hematoxylin and eosin, ×20. (b) Hematoxylin and eosin, ×40. (c) Hematoxylin and eosin, ×200. (d) Immunohistochemical staining by using a monoclonal antibody against human inhibin alpha, ×40 (insert ×200). (e) Immunohistochemical staining by using a monoclonal antibody against human Melan-A, ×40 (insert ×200); a different chromogene was used during developing positive signals. (f) Immunohistochemical staining by using a monoclonal antibody against human Calretinin, ×40 (insert ×200).

A 56-year-old woman without clinical or laboratory evidence of adrenal dysfunction, gravida 4, para 2, was admitted for bilateral salpingo-oophorectomy due to adnexal masses. Grossly, bilateral multiloculated ovarian cysts were identified, which were diagnosed as serous cystadenomas. During microscopic assessment, a 0.2 cm round nodule was found in the adipose tissue adjacent to the hilum of the right ovary. Histologically, it was encapsulated with a thin layer of fibrous tissue and was composed of cells cytologically similar but not identical to those found in Case A (Table 1). There was a well-demarcated zonation pattern in Case B, showing a discontinued subcapsular layer, a layer of cells with clear spongy cytoplasm, a layer of cells with abundant eosinophilic granular cytoplasm, and a central irregular zone of clear cells mixed with thin-walled small vessels. Immunohistologically, the cells were also focally positive for Calretinin, HMB45, and CD10 in addition to showing immunoreactivity with antibodies against inhibin alpha, Melan-A, and Cam5.2 as in Case A. inhibin alpha was predominately positive in the cells with abundant eosinophilic granular cytoplasm (Figure 2(d)). The Melan-A immunostain was diffusely positive while the staining intensity was stronger in subcapsular cells (Figure 2(e)). The immunostaining pattern of Calretinin was similar to that of inhibin alpha but with both nuclear and cytoplasmic staining, which was predominately positive in the cells with abundant eosinophilic granular cytoplasm (Figure 2(f)). The histological and immunological findings supported that the nodule was heterotopic mature adrenocortical tissue (Figure 2).

## 4. Discussion

The developments of the urogenital and adrenal glands are closely associated. Therefore, accessory or heterotopic adrenal cortical tissue is usually found in the retroperitoneal regions near the adrenal glands, kidneys, or along the descending tracts of the urogenital organs [18, 19]. Less common locations of adrenocortical heterotopia have been reported, such as within renal parenchyma [1–3], inside distant organs [4–11], and in the placenta [12–16]. Nonplacental origin of the heterotopia, including those near the urogenital tract and from distant organs, can be seen in any demographically diverse patient populations, adults, children, fetuses, and in both males and females. The lesion is most commonly found as an incidental finding in pediatric patients during inguinoscrotal operations. The reported incidence ranges from 1.6% to 2.5% in these patients [20–22]. Adrenocortical heterotopia is often solitary. Twin nodules next to each other were reported in the perigonadal location of a fetus [23]. Pure adrenocortical cells are demonstrated in all cases except one in a paratracheal location that was reported to have both cortical and medullary components [4].

Adrenocortical heterotopia in the placenta is uncommon. Only six cases have been reported since Cox et al. described the first case [12–16].  Table 2 summarizes the findings of previously reported cases and the current case. In all cases, the heterotopias are from third trimester or term placentas of child-bearing-age women as incidental findings. Two cases are associated with twin placentas [15, 16]. The lesions are all solitary, ovoid, or round and range from less than 0.1 to 0.4 cm (average, 0.2 cm) in diameter. They can be located within either a stem villus, a subchorionic anchoring villus, or a subchorionic space immediately underneath the roof plate, as in the current case. Based on the description of previous reports and again as shown in the current case, the histological features of the adrenocortical nodules from placental locations are similar and show sheets of ovoid or polygonal cells with prominent cell membranes, clear to eosinophilic granular cytoplasm and rich in tiny vessels. In cytology, these cells appear to be similar to those in the zona fasciculate. However, the overall configuration is patternless, and the typical zonation pattern of the adrenal cortex is not seen. It is interesting to note that the typical zonation pattern is also not seen in lesions within solid organs (Table 3), including intrarenal locations [1–3] or within distant organs [4–11]. For instance, the zonation pattern was not shown in an intrahepatic adrenocortical heterotopia in a 38-week neonate, though irregular islands of definitive zone type cortical cells were present [8]. In contrast, the zonation pattern is histologically and immunohistologically seen in Case B, in which the lesion was in close proximity to the ovary. This growth pattern that typically shows three layers of adrenocortical cells is usually seen in other reported cases found close proximity to urogenital organs. In the fetus, the zonation pattern is demarcated by orderly, arranged cells with definitive and fetal zones, reported in a heterotopic adrenocortical nodule of a perigonadal location [23]. Immunohistochemically, the zonation pattern is seen in Case B that is composed of well-differentiated mature adrenocortical tissue, while this is not shown in Case A, which is composed of poorly differentiated and patternless fetal-type-like adrenocortical tissue but lacks zonation growth pattern or a definitive zone. One may question if these cells are functional. A direct answer is yes, since the lesional cells express dehydroepiandrosterone sulfate (DHEA-S) [14] and inhibin alpha. But no evidence has thus far indicated that the heterotopic adrenal gland located in the placenta can undergo a hyperfunctional or neoplastic change. In contrast, heterotopic adrenocortical nodules from nonplacental locations are evident by a variety of pathological changes that lead to hyperfunction, such as hyperplasia, adenoma, or even carcinoma (Table 3). The differences in histology, immunohistochemistry, and functional potential indicate the cellular origins of heterotopic adrenocortical nodules among different locations could be different.

Abnormally located adrenal tissue was once classified into ectopic (accessory) type and heterotopic type [4]. The former, also known as adrenocortical rest, usually denoted those found in the vicinity of the adrenal glands and along the routes of gonadal descent. The latter was named because they were located within other organs. In the most recent surgical pathology textbook by Dr. Rosai [24], adrenocortical heterotopia is used as a general term that includes all abnormally located adrenocortical tissue. Indeed, the terminology is not important in such cases since they are basically similar in the pathological manifestation and the histogenesis is largely unclear. Regarding the histogenesis of these lesions, one hypothesis is that cells forming adrenocortical heterotopia derive from the adrenal primordial stage, then migrate to adjacent or distant organs. The cells of primordial stage in fact function as mobile adrenocortical stem cells. We think that adrenocortical heterotopia may also form locally. This may be true, especially for those in the placental location that can originate from the placental parenchyma or fetal blood. This hypothesis not only explains why adrenocortical tissue is present in organs other than the placenta, but also explains why tissues other than adrenocortex can be found in the placenta [25–27]. In particular, recent studies have demonstrated that placental-derived mesenchymal stem cells (PD-MSCs) are multipotent, which allows them to differentiate into different mesodermal lineage cells such as osteocytes, chondrocytes, cardiac muscle, or endothelial cells [28, 29]. In addition, adrenocortical heterotopia may also be related to genetic or epigenetic alterations. Steroidogenic factor Ad4BP/SF-1, encoded by the *NR5A1* gene in chromosome 3q11, is critical for early adrenal differentiation [30], and transgenic expression of Ad4BP/SF-1 in fetal adrenal progenitor cells results in adrenocortical heterotopia [31]. The results suggest that any genetic alteration or epigenetic signals that lead to Ad4BP/SF1 activation could facilitate the formation of adrenocortical heterotopia.

In summary, we believe that adrenocortical heterotopia of any location is a result of multifactorial reasons that might have genetic and epigenetic roots. What term should be used in diagnosing an abnormally located adrenocortical tissue is probably not of great concern. For clinical practice, the key issue is to make the correct diagnosis and to understand that the majority of such lesions are clinically insignificant.

## Figures and Tables

**Table 1:** Clinical and pathological characteristics of reported cases.

Findings	Case A	Case B
Patient	30 yo female	56 yo female
Location	Term placenta, subchorionic	Right ovary, hilar fat
Lesion size	0.2 cm	0.2 cm
Shape	Round nodule	Round nodule
Capsule	Pseudocapsule	Thin fibrous capsule
Zonation pattern	No	Yes
Vasculature	Abundant	Less abundant
Differentiation	Immature	Mature

**Table 2:** Summary of basic clinical and pathological features of adrenocortical heterotopia in the placenta.

%	Maternal age	Gestational age	Placenta (gram)	Lesion size (cm)	Location	Reference
1	27 (g2p1)	36	?	?	Stem villus	[12]
2	25 (g3p2)	?	350	0.2	Stem villus	[13]
3	26 (g2p1)	37	540	0.4	Stem villus	[14]
4	30 (g2p1)	34 (twin)	405/340	<0.1	Stem villus	[15]
5	39 (g9p7)	38	350	<0.1	Stem villus	[15]
6	29 (g1p1)	? (twin)	280/285	0.2	Subchorionic	[16]
7	30 (g3p2)	40	560	0.2	Subchorionic	Current case

**Table 3:** Comparison of basic pathological features of adrenocortical heterotopia of different locations.

	Vicinity of urogenital organs	Intrarenal or within distant organs	Placental
Zonation pattern	Yes	No	No
Medullar component	Not reported	Reported in one case [4]	Not reported
Shape	Round	Round or irregular	Round
Size	Variable	Up to 1.5 cm	~0.2 cm
Number of lesions	Two lesions reported in one fetus [22]	Solitary	Solitary
Hyperfunctional or neoplastic	Reported	Reported	Not reported
References	[17, 18, 22]	[1–11]	[12–16]
